# Treatment Response Prediction Using Ultrasound-Based Pre-, Post-Early, and Delta Radiomics in Neoadjuvant Chemotherapy in Breast Cancer

**DOI:** 10.3389/fonc.2022.748008

**Published:** 2022-02-07

**Authors:** Min Yang, Huan Liu, Qingli Dai, Ling Yao, Shun Zhang, Zhihong Wang, Jing Li, Qinghong Duan

**Affiliations:** ^1^ Department of Medical Imaging, the Affiliated Tumor Hospital of Guizhou Medical University, Guiyang, China; ^2^ Department of Advanced Application Team, GE Healthcare, Shanghai, China; ^3^ Department of Breast Surgery, the Affiliated Tumor Hospital of Guizhou Medical University, Guiyang, China; ^4^ Department of Medical Imaging, The Affiliated Hospital of Guizhou Medical University, Guiyang, China

**Keywords:** nomogram radiomics, ultrasound, neoadjuvant chemotherapy, breast cancer, Ki-67

## Abstract

**Objective:**

To develop and validate a radiomics nomogram based on pre-treatment, early treatment ultrasound (US) radiomics features combined with clinical characteristics for early prediction of response to neoadjuvant chemotherapy (NAC) in breast cancer.

**Method:**

A total of 217 patients with histological results of breast cancer receiving four to eight cycles of NAC before surgery from January 2018 to December 2020 were enrolled. Patients from the study population were randomly separated into a training set (n = 152) and a validation set (n = 65) at a ratio of 7:3. A total of 788 radiomics features were extracted from each region of interest in the US image at pre-treatment baseline (radiomic signature, RS1), early treatment (after completion of two cycles of NAC, RS2) and delta radiomics (calculated between the pre-treatment and post-treatment features, Delta RS). The Max-Relevance and Min-Redundancy (mRMR) and the least absolute shrinkage and selection operator (LASSO) regression were applied for feature selection. The predictive nomogram was built based on the radiomics signature combined with clinicopathological risk factors. Discrimination, calibration, and prediction performance were further evaluated in the validation set.

**Results:**

Of the 217 breast masses, 127 (58.5%) were responsive to NAC and 90 (41.5%) were non-responsive. Following feature selection, nine features in RS1, 11 features in RS2, and eight features in Delta RS remained. With multivariate analysis, the RS1, RS2, Delta RS, and Ki-67 expression were independently associated with breast NAC response. However, the performance of the Delta RS (AUC*_Delta RS_* = 0.743) was not higher than RS1 (AUC*_RS1_ =* 0.722, *P_Delta *vs* RS1_* = 0.086) and RS2 (AUC*_RS2_ =* 0.811, *P_Delta *vs* RS2 =_* 0.173) with the Delong test. The nomogram incorporating RS1, RS2, and Ki-67 expression showed better predictive ability for NAC response with an area under the curve (AUC) of 0.866 in validation cohorts than either the single RS1 (AUC 0.725) or RS2 (AUC 0.793) or Ki-67 (AUC 0.643).

**Conclusion:**

The nomogram incorporating pre-treatment and early-treatment US radiomics features and Ki-67 expression showed good performance in terms of NAC response in breast cancer, thereby providing valuable information for individual treatment and timely adjustment of chemotherapy regimens.

## Introduction

Breast cancer is one of the most common cancers in women and is the leading overall cause of cancer-related death in females worldwide (1). Neoadjuvant chemotherapy (NAC), which was proposed by Frei in 1982, refers to the systemic cytotoxic drug treatment for non-metastatic tumors before radical surgery or radiotherapy (2). For breast cancer, preoperative NAC is likely to reduce the clinical stage and tumor size and alleviate lymph node metastasis. The treatment can improve the rate of breast-conserving surgery for patients with resectable surgery and create an opportunity for surgery for patients whose tumors cannot be removed surgically, hence becoming the standard treatment for selected high-risk breast cancers such as tumors ≥2 cm in size and for locally advanced disease (namely, tumors initially ineligible for resection) (3). However, not all patients benefit from NAC, and those who fail to respond to treatment face the risk of delayed surgery and aggravated disease. Early prediction of patient treatment response can timely adjust the chemotherapy regimen and avoid patients suffering from severe toxic and side effects from NAC.

In current clinical practice, the effect of NAC is commonly evaluated by medical imaging techniques and immunohistochemistry (IHC). The evaluation of pathologic complete response (pCR) of breast tumors could accurately reflect real changes in lesions, but pCR evaluation can only be performed after radical surgery, and preoperative NAC cannot be timely adjusted (4). In addition, it has been suggested that the response evaluation criteria in solid tumor (RECIST) be used to record changes in the maximum diameter of breast cancer lesions before and after NAC to evaluate its efficacy, but this strategy can only be evaluated after the chemotherapy cycle is completed and visual interpretations are generally subjective and may lead to mistakes at the cut-off values (5).

With the rapid development of fractionated biological techniques, the expression of a few biomarkers may be associated with the prediction of response to NAC at the time of initial diagnosis. A recent meta-analysis of the relationship between the expression level of Ki-67 before NAC showed that the pCR rate of patients in the Ki-67 high-expression group was 3.1-fold that of the low expression group. Ki-67 is an indicator of cell proliferation and thus may be utilized as a predictor and prognostic index of NAC efficacy in breast cancer (6, 7). However, a series of factors, namely, heterogeneous patient populations, small sample size, poor representativeness of the tumor tissues derived from core biopsies, and different NAC regimens may lead to the inability of Ki-67 to accurately predict the response to NAC (8).

In the digital era, recent studies have illustrated that tumor characteristics at the genetic and cellular levels can be captured from medical images by high-throughput feature extraction and computation. Radiomics involves the extraction and analysis of large amount of quantitative imaging features to provide a comprehensive characterization of entire tumors rather than from a relatively limited tissue sample and can reveal predictive or prognostic associations between images and medical outcomes (9, 10). Ultrasonography, based on its broad adaptability, low cost, non-radiation, and rapid and non-invasive features, is essential for the management of breast cancer from diagnosis, staging, and treatment planning to postoperative surveillance and prognosis of malignant tumor evaluation. The China Anti-Cancer Association breast cancer guidelines strongly recommend that patients be re-evaluated for therapy response regularly using ultrasound (US) after each two cycles of NAC (11). However, visual assessment and qualitative descriptions for US features mainly depend on the personal clinical experience and subjective judgment of doctors, which inevitably leads to inter-observer variability and subjective diagnostic decision-making assessment of tumor heterogeneity (12, 13). The integration of radiomics features, namely, first-order statistics, texture, and wavelet features extracted from the US images can significantly increase the objectiveness of image diagnosis. A radiomics methodology has been more recently applied to distinguish benign malignant breast lesions or predict lymph node status, prognosis, and even treatment response (14, 15). However, most of the current studies focus on the analysis of MRI or CT images, and a few studies have reported radiomic features of breast masses analyzed using breast US. More importantly, due to the high heterogeneity and complexity of the internal structure of malignant tumors, the imaging characteristics before and after chemotherapy have obvious differences in microstructure. Studies have shown that pre-treatment initial images are often related to primary tumor characteristics, while images after chemotherapy can reflect the active state of the tumor. Extracting the high-order imaging radiomic features and analyzing the differences in features before and after treatment, namely, delta radiomics, can more accurately evaluate sensitivity to chemotherapy (16).

The purpose of this study was to develop a radiomics signature based on first-order statistics, textural, and wavelet features from breast cancer US images at pre-treatment and early treatment and also the corresponding delta radiomics of NAC, and further develop a multi-parameter radiomics prediction model to evaluate the efficacy predictive value of the model for NAC of breast cancer. This strategy is expected to achieve early assessment of NAC therapy-responsive vs. -unresponsive in breast cancer from the routine US images, which can provide more accurate prediction performance and obviate the need for chemotherapy in select settings.

## Materials and Methods

### Patients

**Figure 1:**
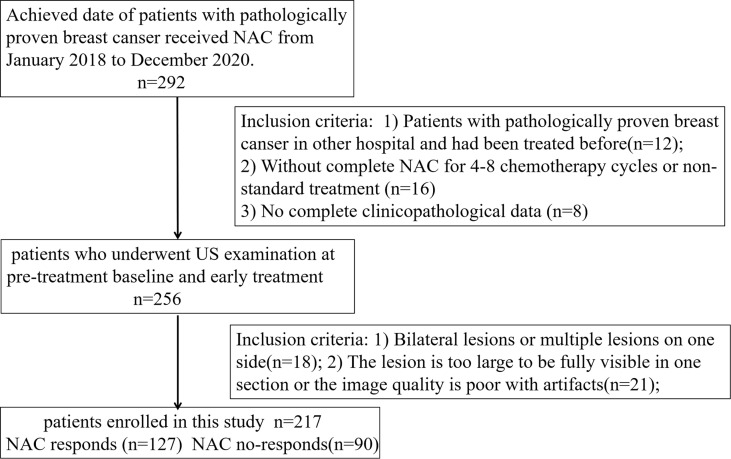
Flowchart shows study population and exclusion criteria.

Ethical approval for this retrospective study was obtained from our institutional review board, and informed consent was waived. A total of 217 patients with breast cancer confirmed by pathology in the Affiliated Cancer Hospital of Guizhou Medical University from December 2018 to December 2020 were selected. **Figure 1** shows the identification, eligibility, and inclusion of patients in the study. The inclusion criteria were: 1) Patients with invasive breast carcinoma confirmed by histopathology from biopsy in the hospital for the first time who had not been treated before; 2) Patients received the NAC regimen based on paclitaxel and anthracycline drugs for four to eight chemotherapy cycles; 3) US examination was performed at pre-treatment baseline (radiomics signature, RS1) and early treatment (after completion of the two cycles of chemotherapy, RS2). The exclusion criteria included the following: 1) Bilateral lesions or multiple lesions on one side; 2) The lesion was too large to be fully visible in one section or the image quality was poor with artifacts; and 3) No complete clinicopathological data or US images. The clinical and histopathological data were collected from patient medical records and included age, histological type, clinical staging, molecular subtype, and proliferation marker Ki-67 expression, which were recorded by noting the percentage of positively stained malignant cells. The Ki-67 level was classified as positive when the threshold value was ≥14%, whereas it was classified as negative when the threshold value was <14% (17, 18). Then, patients from the study population were grouped using randomly stratified sampling in a ratio of 7:3 to obtain a training set (n = 152; 89 responders, 63 non-responders) and a validation set (n = 65; 38 responders, 27 non-responders).

Participants were then classified as responders (R) or non-responders (NR) based on the radiological evaluation by the RECIST-based criteria in tumor size reduction. The size of breast lesions was evaluated by two radiologists with more than five years of working experience, where each of them was performed twice for interobserver and intraobserver reproducibility and the error range of measurement of the two radiologists is within 5 mm. The response standard of RECIST is a percent reduction in tumor size (in its longest dimension) between pre-treatment and post-treatment times of at least 30% (19), and in order to avoid single examination measurement error, the tumor size was determined by ultrasound and MRI image. In this study, a patient was described as R when the tumor size of both ultrasound and MRI measurements all reduced by at least 30% compared to the pre-treatment dimensions, and other patients being considered as NR.

### US Examination and Image Feature Analysis

All patients were examined using a Logiq E9^®^ scanner (GE Healthcare, Wauwatosa, WI, USA) equipped with a linear array transducer having a central frequency of 7 MHz. Ultrasonography was performed at pre-treatment baseline and at early treatment (after completion of the two cycles of chemotherapy). According to the characteristics of breast lesions, appropriate frequency, depth, focus, gain, and time gain compensation curves were selected to achieve the best grayscale US imaging quality. According to alder grade, the blood flow signal with the highest resistance index (RI) in the lesion was collected, and the average value of three times was taken (20). The grayscale images with the largest long-axis cross-section image were saved in DICOM format.

Then, the lesion was evaluated according to the second edition of the 2013 US Breast Imaging Report and Data Analysis System (BI-RADS) by the two radiologists with more than five years of working experience without knowing any clinical data and pathological results (21). The grayscale ultrasonographic features of tumors, namely, lesion maximum diameter, internal echo, micro-calcification, morphology, blood flow grade (grades 1 and 2 were classified as hypovascular, while grades 3 and 4 were classified as hypervascular), and RI were recorded in detail.

### Image Segmentation and Feature Extraction

**Figure 2:**
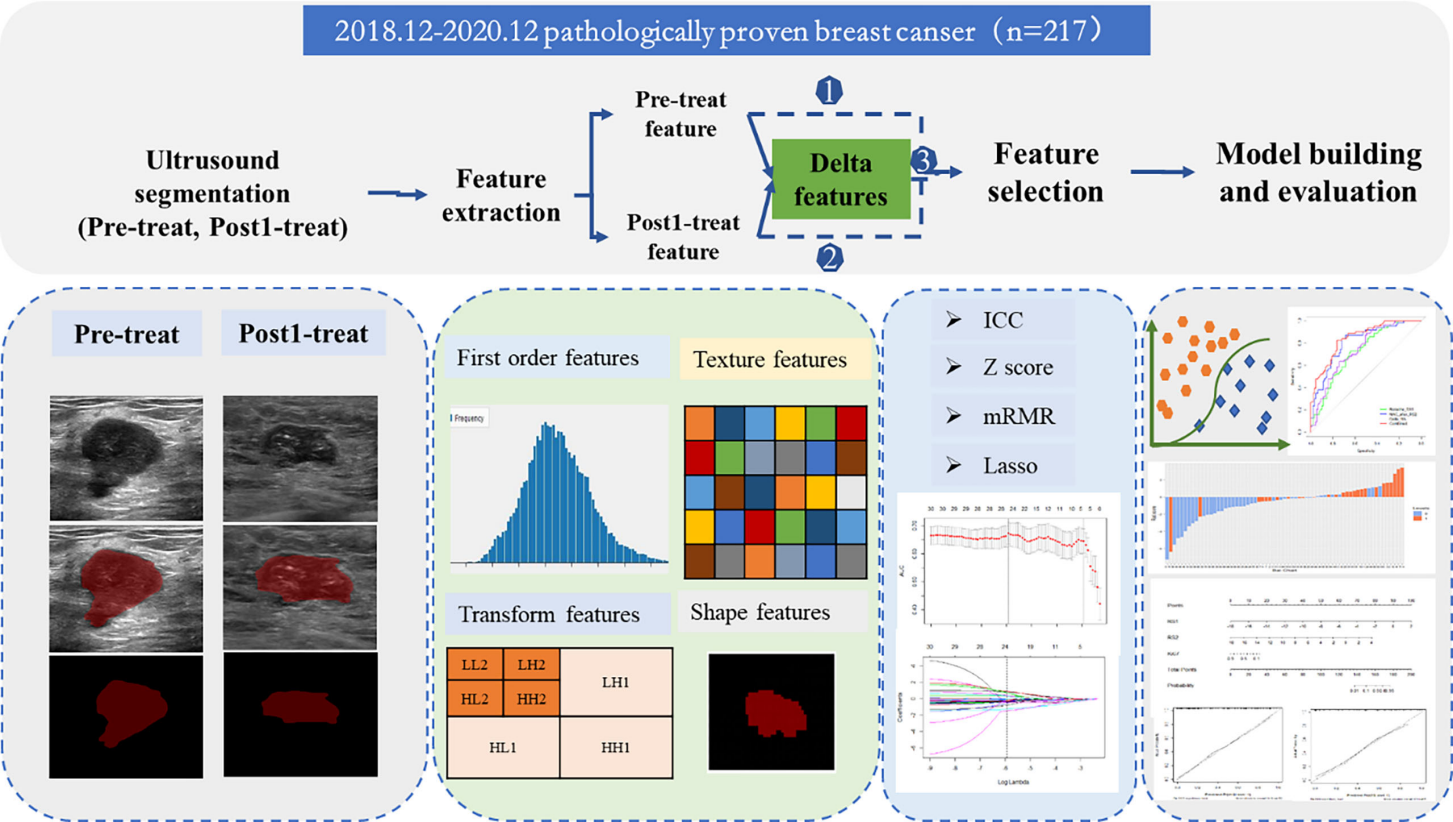
A flowchart of the processing step using the radiomic method for predicting NAC response.

A flowchart of the processing step using the radiomics method for predicting NAC responses is shown in **Figure 2**. The image of pre-NAC and early post-NAC US imaging per lesion were used for analysis. A region of interest (ROI) was manually drawn around the boundary of the index mass on the grayscale image by a >5 year-experienced radiologist using the ITK-SNAP software (http://www.itksnap.org/). A two-dimensional ROI of the tumor before NAC (ROI-1) and after NAC (ROI-2) was delineated on the US images. Fifty patients were randomly selected for the radiologist to perform the second segmentation after 1 week. Then, the ROI segmented images were imported into the USKit software (ultrasound kit, version 1.3.0, GE Healthcare) for automatic feature extraction, namely, first-order features, shape features, texture features, and transform features. First-order features mainly described the distribution of voxel intensities within the lesions in the CT image; shape features mainly described the geometric and shape characteristics of the lesions in the CT image; and texture features based on gray-level co-occurrence matrix (GLCM) and gray level run length matrix (GLRLM) mainly described the texture with the spatial relationship between the distance and angle of different pixel pairs. To enhance intricate patterns in the data invisible to the human eye, advanced filters, including wavelet decompositions with all possible combinations of high (H) or low (L) pass filters in each of the three dimensions (HHH, HHL, HLH, LHH, LLL, LLH, LHL, and HLL), were applied.

The radiomic features of pre-NAC and early post-NAC US imaging were directly obtained from the pre-mapped ROI, and the delta radiomic features were defined as follows: *Delta _feature_= (Early treatment_features_ − Baseline_feature_)*/*Baseline_feature_. *A total of 1,576 radiomics features of each patient (788 features at each time point) were finally extracted, and the corresponding of 788 delta radiomic features were obtained at the same time.

Intraclass correlation coefficient (ICC) was used to evaluate the consistency of evaluation results among physicians. The level of clinical significance evidence of the ICC was judged by the previous similar ultrasound radiomics study: an ICC of <0.40 was rated as ‘Poor’, ICC of 0.40–0.59 as ‘Fair’, ICC of 0.60–0.74 as ‘Good’ and ICC of 0.74–1.00 as ‘Excellent’ (22).

### Feature Selection

Before the feature selected process, the abnormal and missing values were replaced by the median, the ICC was calculated to ensure repeatability and stability of features with the threshold of 0.7. Max-Relevance and Min-Redundancy (mRMR) and the least absolute shrinkage and selection operator (LASSO) regression were used to select significant features (23). Then, a radiomics score was calculated using a formula incorporating the selected features that were weighted by their respective coefficients.

### Development of the Prediction Model

The prediction model for predicting tumor responses to NAC were developed using data from the training group. First, the univariate analyses were performed to select the significant factors, including clinical features and grayscale US features. Then, single-factor statistically significant indicators with P-values <0.05 were included in multivariate logistic regression analysis, and factors with P-values <0.05 were considered independent predictors after the multivariate analysis. Finally, the selected predictive features were incorporated with a radiomics signature to establish logistic regression models and verify the stability of the constructed model by 10-fold cross-validation in the training group. For comparison, a clinical model only comprising significant clinical information was constructed. In addition, Receiver operating characteristic (ROC) curves were plotted with the optimal cut-off value that was defined as maximizing the Youden index (Sensitivity + Specificity − 1). Bar diagrams were plotted to clearly display the discrimination performance of the nomogram. The area under the curve (AUC), sensitivity, specificity, and accuracy were then calculated using the training set.

### Prediction Performance Evaluation of the Nomogram

The prediction performance of the generated radiomics nomogram and the radiomics and clinicopathological combined nomogram were further tested in the independent validation set using the same cut-off value determined in the training set. The accuracy, sensitivity, specificity, and AUC of the ROC curve were plotted to assess the prediction performance of the nomogram. The Delong test was used to compare the combined model with the other constructed model. The agreement between the observed outcome frequencies and predicted probabilities was assessed using a calibration curve to explore the predictive accuracy of the nomogram (24).

### Statistical Analysis

Statistical analysis was performed using SPSS 22.0 (Chicago, IL, USA) and R software (version 3.6.0; https://www.Rproject.org). An independent *t*-test was used to compare continuous variables with a normal distribution, while the Mann–Whitney U test was used to compare continuous variables with an abnormal or unknown distribution. The χ² test was used to compare categorical variables. A P-value of <0.05 was considered statistically significant. The “mRMRe” and “glmnet” packages were applied to perform mRMR and LASSO, respectively. The ROC curve of each model was plotted using the “pROC” package. The “rms” package was used to build the nomogram.

## Results

### Patient Information and Clinicopathologic Characteristics

The basic information and clinicopathologic characteristics of the research population are summarized in **Table 1**. According to the clinical and pathological criteria assessment, 127 (62.5%) and 90 (37.5%) patients were classified as responders and non-responders, respectively. Univariate analysis revealed several clinical factors that were obviously different (P <0.1), namely, molecular typing and PR status, and Ki-67 status were significantly different (P <0.001) (**Table 1**). A clinical model based on the significant clinical factors for NAC response prediction was constructed using logistic regression analysis. In addition, no statistical difference patient clinical characteristics were observed between the training set and the validation set as shown in **Table 1**.

**Table 1:** Basic information, clinicopathologic characteristics, and two-dimensional general ultrasonic characteristics of the study cohorts.

Variable	Responder (n = 127)	Non-Responder (n = 90)	P-value	Training Cohort (n = 152)	Test Cohort (n = 65)	P-value
Histologic type			0.67			0.288
Invasive ductal carcinoma	120 (94.49%)	87 (96.67%)		147 (96.71%)	60 (92.31%)	
Others	7 (5.51%)	3 (3.33%)		5 (3.29%)	5 (7.69%)	
Molecular subtyping			0.076’			0.243
Luminal A	6 (4.72%)	12 (13.33%)		16 (10.53%)	2 (3.08%)	
Luminal B	56 (44.09%)	43 (47.78%)		65 (42.76%)	34 (52.31%)	
Her2+	48 (37.80%)	24 (26.67%)		52 (34.21%)	20 (30.77%)	
Triple-negative	17 (13.39%)	11 (12.22%)		19 (12.50%)	9 (13.85%)	
Tumor stage			0.141			0.471
I	5 (3.94%)	4 (4.44%)		6 (3.95%)	3 (4.62%)	
II	60 (47.24%)	40 (44.44%)		65 (42.76%)	35 (53.85%)	
III	49 (38.58%)	27 (30.00%)		57 (37.50%)	19 (29.23%)	
IV	13 (10.24%)	19 (21.11%)		24 (15.79%)	8 (12.31%)	
Histologic grade			0.623			0.385
I	42 (33.07%)	24 (26.67%)		42 (27.63%)	24 (36.92%)	
II	82 (64.57%)	63 (70.00%)		105 (69.08%)	40 (61.54%)	
III	3 (2.36%)	3 (3.33%)		5 (3.29%)	1 (1.54%)	
ER			0.173			0.959
Positive	79 (62.20%)	64 (71.11%)		100 (65.79%)	43 (66.15%)	
Negative	48 (37.80%)	26 (28.89%)		52 (34.21%)	22 (33.85%)	
PR			0.068’			0.62
Positive	69 (54.33%)	60 (66.67%)		92 (60.53%)	37 (56.92%)	
Negative	58 (45.67%)	30 (33.33%)		60 (39.47%)	28 (43.08%)	
Her2			0.431			0.569
Positive	36 (28.35%)	30 (33.33%)		48 (31.58%)	18 (27.69%)	
Negative	91 (71.65%)	60 (66.67%)		104 (68.42%)	47 (72.31%)	
Ki-67			<0.001*			0.578
>14%	77 (60.63%)	47 (52.22%)		67 (44.08%)	26 (40.00%)	
<14%	50 (39.37%)	43 (47.78%)		24 (15.79%)	10 (15.38%)	
Tumor_internal_echo						0.094
Uniform	20 (15.75%)	14 (15.56%)	0.969	24 (15.79%)	10 (15.38%)	
Non-uniform	107 (84.25%)	76 (84.44%)		128 (84.21% %)	55 (84.62%)	
Micro_calcification						0.442
Yes	46 (36.22%)	29 (32.22%)	0.542	55 (36.18%)	20 (30.77%)	
No	81 (63.78%)	61 (67.78%)		97 (63.82%)	45 (69.23%)	
Morphology						0.387
Regular	17 (13.39%)	13 (14.44%)	0.824	19 (12.50%)	11 (16.92%)	
Irregular	110 (86.61%)	77 (85.56%)		133 (87.50%)	54 (83.08%)	
Blood_flow_grade						0.365
Grades 1–2	25 (19.69%)	20 (22.22%)	0.65	34 (22.37%)	11 (16.92%)	
Grades 3–4	102 (80.31%)	70 (77.78%)		118 (77.63%)	54 (83.08%)	
Age	50.00 (44.00, 57.00)	49.00 (42.00, 55.05)	0.384	49.50 (44.00, 56.00)	49.00 (43.70, 57.00)	0.954
max_D_Baseline	28.00 (23.00, 37.80)	25.00 (15.00, 33.15)	0.008*	26.00 (16.45, 38.00)	26.00 (21.70, 33.30)	0.809
max_D_NAC	15.00 (10.00, 22.00)	22.00 (15.95, 32.00)	<0.001*	17.50 (12.00, 27.55)	19.00 (13.00, 24.30)	0.811
RI	0.78 (0.72,0.84)	0.78 (0.73, 0.83)	0.843	0.78 (0.72,0.83)	0.80 (0.73, 0.85)	0.174

Chi-square test or Fisher’s exact test was used for the nominal variable, and Mann–Whitney test was used for the continuous variable with abnormal distribution. A two-tailed p-value <0.05 indicated statistical significance.

*Indicates a statistical difference between the Responder group and Non-Responder group.

### Comparison of Grayscale Ultrasonographic Features

The results of comparison of grayscale ultrasonographic features of the clinical response group to the non-response group at pre-treatment baseline and at early treatment are shown in **Table 1**. The responder group demonstrated a statistically significant reduction in maximum tumor size from 28 mm prior to treatment compared to 15 mm after early treatment (P <0.05). Except for the maximum tumor size, tumor internal echo, microcalcification, morphology, blood flow grade, and RI of the grayscale US image, none of the parameters demonstrated significant differences in terms of distribution between the two groups (**Table 1**). In addition, no significant difference was found between the training set and the validation set. The results of the consistency test showed that the ICC of the two observers was within the range of 0.74–0.91.

### Feature Selection and Radiomic Signature Construction

**Figure 3:**
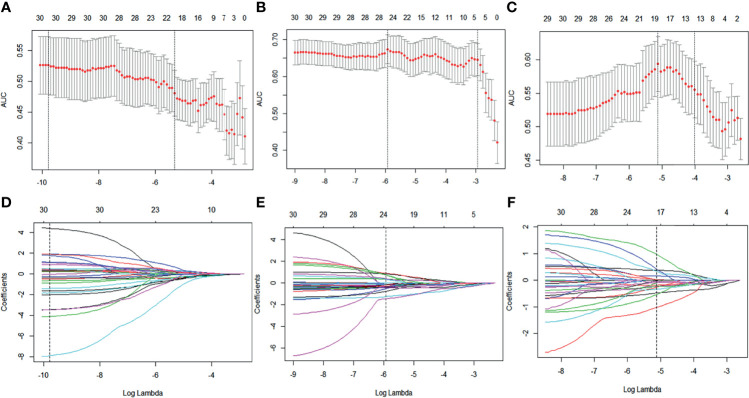
Baseline, early treatment, and delta radiomics feature selection by LASSO regression. **(A–C)** Selection of tuning parameters (lambda value) in the LASSO model using 10-fold cross-validation by the minimum criteria. **(D–F)** LASSO coefficient profiles of the radiomics features.

A total of 788 candidate radiomic features were extracted from each ROI. Before selection, the 126 features remained with the ICC (>0.7) to ensure repeatability of features. Next, 30 features were selected with the mRMR. **Figure 3** shows the process of feature selection at baseline, early treatment, and Delta radiomics (baseline minus early treatment) by LASSO regression. Then, 9 features in RS1(pre-treatment baseline), 11 features in RS2 (early treatment), and 8 features in Delta RS (baseline minus early treatment) were selected and the ICC values of each feature are greater than 0.7, specific values are shown in **Figure S1** in the **Supplementary Material**. In addition, the Akaike information criterion was used to evaluate goodness of fit. The selected NAC responder-related features shown in **Table 2** were used to calculate the radiomics score of RS1, RS2, and Delta RS according to their coefficients by the corresponding formula. No statistical differences in the distribution of the radiomics score were found between the training and validation set. The selected significant features were used to establish the radiomics signature and 10-fold cross-validation has verified the stability of the constructed model in the training cohort (the results are shown in **Figure S2** in the **Supplementary Material**).

**Table 2:** Selected features and their coefficients in the model.

Model	Feature	Coefficient	P-value
Baseline	wavelet.HLH_firstorder_Median	4.32e17	0.171
	wavelet.LHH_glcm_Imc2	26.2175	0.008
	wavelet.HHH_firstorder_Kurtosis	−0.0064	0.038
	wavelet.HHL_gldm_SmallDependenceHighGrayLevelEmphasis	−0.00086	0.019
	wavelet.HHH_firstorder_Skewness	0.378728	0.028
	wavelet.HHL_glszm_GrayLevelNonUniformityNormalized	−7.5178	0.0035
	wavelet.HLL_glrlm_RunPercentage	−6.9633	0.022
	wavelet.HLL_firstorder_Median	−22.3364	0.181
	wavelet.LHH_glcm_ClusterProminence	−43.8422	0.015
	intercept	26.10966	
Two cycles after NAC	wavelet.HLL_glszm_LargeAreaLowGrayLevelEmphasis	−0.00017	0.172
	wavelet.HHH_firstorder_Kurtosis	−0.00494	0.085
	wavelet.LHH_glcm_Imc2	−17.0163	0.057
	wavelet.HHL_firstorder_Skewness	0.2961	0.042
	wavelet.HHL_glszm_LargeAreaLowGrayLevelEmphasis	2.80e−05	0.024
	wavelet.HHL_firstorder_Kurtosis	0.0085	0.040
	wavelet.HHL_gldm_LargeDependenceHighGrayLevelEmphasis	−1.48e−5	0.0007
	wavelet.HLL_firstorder_InterquartileRange	0.4055	0.024
	wavelet.HLL_firstorder_Median	−10.2665	0.004
	wavelet.LHH_glcm_ClusterProminence	21.7567	0.119
	wavelet.HHL_glrlm_ShortRunLowGrayLevelEmphasis	−51.5129	0.006
	intercept	−8.50213	
Delta	∆wavelet.LHL_glszm_SmallAreaLowGrayLevelEmphasis	−226.797	0.042
	∆wavelet.LHH_glcm_Imc2	−12.536	0.037
	∆wavelet.HHL_glszm_LargeAreaLowGrayLevelEmphasis	4.47e−6	0.063
	∆wavelet.HHL_firstorder_Kurtosis	0.000994	0.111
	∆wavelet.HLL_firstorder_InterquartileRange	0.4609	0.003
	∆wavelet.HLL_firstorder_Median	−7.304	0.003
	∆wavelet.LHL_gldm_SmallDependenceHighGrayLevelEmphasis	−5.39e−5	0.080
	∆wavelet.LHH_glcm_ClusterProminence	20.9419	0.0425
	intercept	−0.06275	

**Figure 4:**
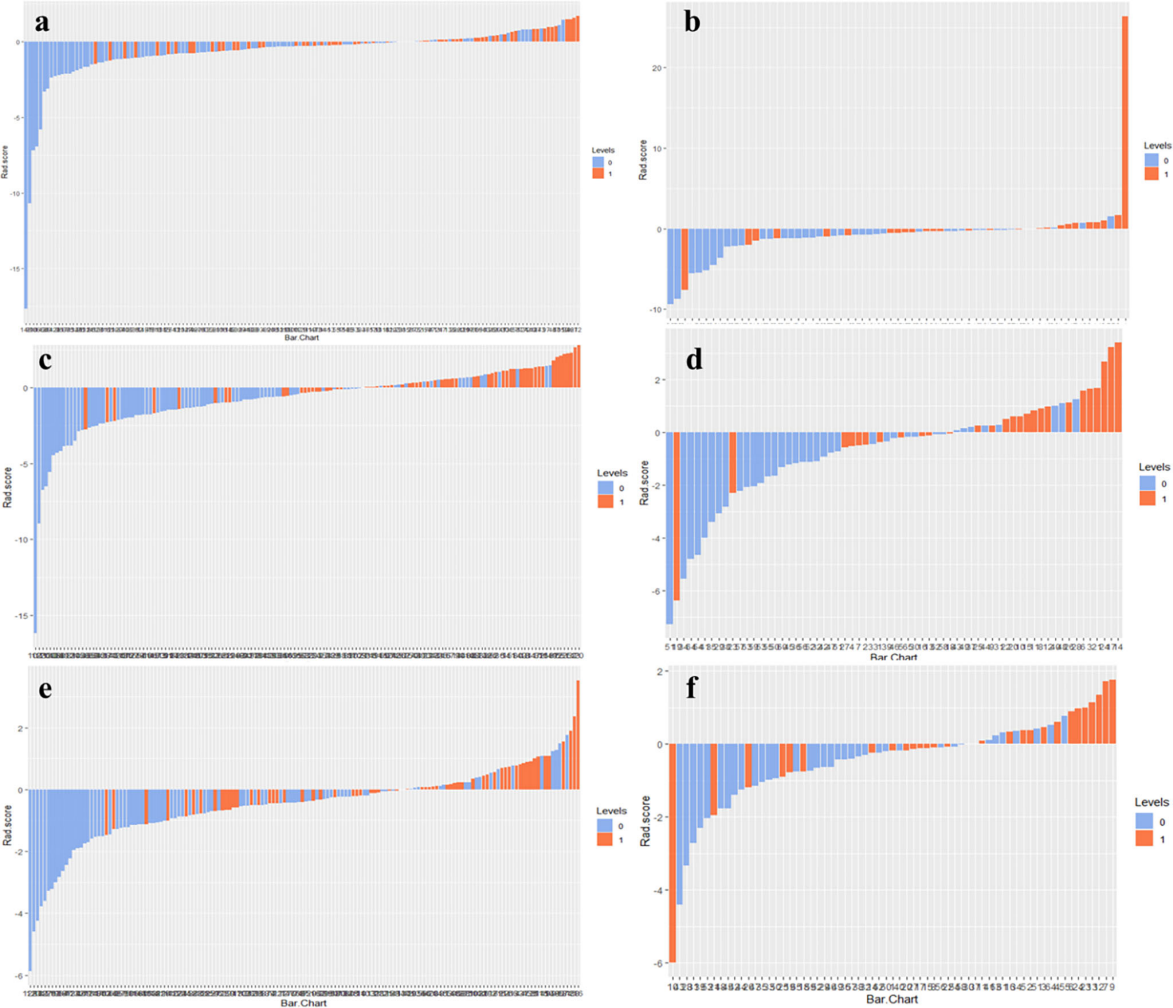
Radiomics score bar diagrams of RS1, RS2, and Delta RS in the training **(A, C, E)** and validation sets **(B, D, F)** were plotted. Up and down bars refer to the predicted NAC responding and NAC non-responding lesions, respectively. Blue and red bars refer to actual NAC responding and NAC non-responding lesions, respectively.

The optimal cut-off values of RS1, RS2, and Delta RS for the radiomics score for discriminating NAC responders and non-responders were −0.298, −0.4343, and −0.6979, respectively, in the training group. Radiomics score bar diagrams were plotted based on this optimal cut-of value in the training and validation set (**Figure 4**). The bar diagrams demonstrated good discrimination performance of the radiomics score.

### Development and Validation of the Radiomics Nomogram

**Figure 5:**
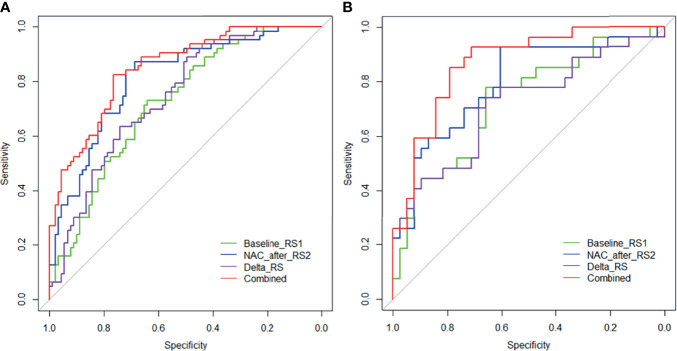
Receiver operating characteristic (ROC) curves of the RS1 (green lines), RS2 (blue lines), Delta RS (purple lines), and nomogram (red lines) in the training **(A)** and validation **(B)** groups.

The performance of the RS1, RS2, Delta RS, and nomogram for predicting NAC responders in the training and validation groups is shown in **Table 3**, and the ROC curves of different model in both groups are presented in **Figure 5**. The prediction performance of the RS1, RS2, and Delta radiomics models was moderate, with AUC values of 0.722 (95%CI [0.643, 0.802]), 0.811 [95%CI (0.742, 0.880)], and 0.743 [95%CI (0.666, 0.820)], respectively, in the training cohort and 0.725 [95%CI (0.543, 0.814)], 0.793 [95%CI (0.679, 0.908)], and 0.714 [95%CI (0.582, 0.847)] in the validation cohort, respectively, and Ki-67 with an AUC of 0.643 [95%CI (0.575, 0.706)]. However, the performance of the Delta RS (AUC*_Delta RS_* = 0.743) was not higher than the RS1 (AUC*_RS1_ =* 0.722, *P_Delta *vs* RS1_* = 0.086) and RS2 (AUC*_RS2_ =* 0.811, *P_Delta *vs* RS2 =_* 0.173) with the Delong test. The nomogram that incorporated RS1, RS2, and Ki-67 displayed AUC values of 0.849 (95%CI [0.789, 0.908]) for predicting NAC responders in the training cohort, and the accuracy, sensitivity, and specificity were 0.750, 0.825, and 0.764, respectively. In the validation cohort, it also displayed excellent prediction efficacy, with an AUC of 0.866 (95%CI [0.779, 0.954]), and the accuracy, sensitivity, and specificity were 0.785, 0.852, and 0.798, respectively.

**Table 3:** Comparison of different models.

Model	Training Cohort (n = 152)				Test Cohort (n = 65)				Delong
	AUC (95%CI)	Sen.	Spec.	ACC	AUC (95%CI)	Sen.	Spec.	ACC	
RS1 (Baseline)	0.722 (0.643–0.802)	0.730	0.640	0.678	0.725 (0.543–0.814)	0.778	0.658	0.677	0.971
RS2 (NAC_after two cycles)	0.811 (0.742–0.880)	0.719	0.841	0.750	0.793 (0.679–0.908)	0.605	0.926	0.723	0.795
Delta RS	0.743 (0.666–0.820)	0.494	0.889	0.678	0.714 (0.582–0.847)	0.658	0.741	0.692	0.717
Nomogram	0.849 (0.789–0.908)	0.825	0.764	0.750	0.866 (0.779–0.954)	0.852	0.789	0.785	0.742

Sen., sensitivity; Spec.,specificity; ACC, accuracy.

**Figure 6:**
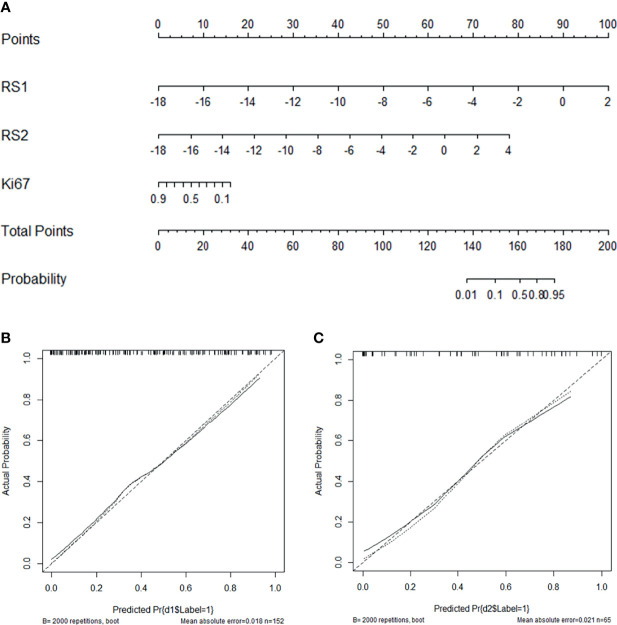
Nomogram with the RS1, RS2, and Ki-67 incorporated **(A)** and calibration curves for the nomogram in the training **(B)** and validation groups **(C)**.

**Table 4** displays the results of multivariable logistic regression analysis of risk factors for NAC responders in the training group. RS1, RS2, and Ki-67 expression states were demonstrated to be significant for NAC responders (P <0.001), and the nomogram is shown in **Figure 6A**. The calibration curves (**Figures 6B, C**) in the training and validation groups were tested using the Hosmer–Lemeshow test and yielded a non-significant difference in training cohorts (P = 0.11) and validation cohorts (P = 0.75), respectively. The nomogram showed good agreement in predicting NAC responders in advance compared to the comprehensive evaluation of radiological and pathological conformation after completing NAC treatment. In addition, the Delong test was applied to test the significance among the models (nomogram vs. RS1 <0.001, nomogram vs. RS2 = 0.376).

**Table 4:** Multivariable logistic regression analysis of risk factors of NAC responders.

Characteristic	β	Odds Ratios (95%CI)	P-value
Ki-67	−3.455	0.0316 (0.004–0.244)	<0.001*
RS1	0.9715	2.642 (1.497–4.661)	<0.001*
RS2	0.6891	1.992 (1.429–2.775)	<0.001*
Constant	1.715		

*Indicates a statistical difference between the Responder group and Non-Responder group.

## Discussion

In the present study, a US image-based radiomics score was built and validated as a pre-treatment independent predictor of NAC response in patients with breast cancer. Then, we developed a nomogram based on pre-treatment and early-treatment US data and significant clinical characteristics (Ki-67 expression) to predict NAC response. The nomogram displayed an excellent ability to predict NAC response, with AUC values of 0.849 and 0.866, and accuracy values of 0.750 and 0.785 in the training and validation cohorts, respectively. The calibration curve showed that the predicted and actual result of NAC were in good agreement. The outperformance of the nomogram indicated that combining high-throughput digital US features with Ki-67 expression could be helpful for the early individualized prediction of efficiency to NAC in breast cancer, which might provide useful information for clinical decision-making.

Pathological evaluation, as the gold standard for breast cancer NAC curative effect evaluation, has a lag, and the treatment plan cannot be adjusted in a timely manner according to the progress of the condition of the patient. Current methods for assessing responses to NAC mostly use conventional breast imaging, namely, X-ray, US, and MRI. However, most of these imaging examinations mainly depend on changes in mass size to make therapeutic judgments and predictions, and it is very difficult to accurately predict the efficacy of NAC before or during chemotherapy (25, 26). The results of this study also showed that the characteristics of 2-D US, including tumor internal echo, microcalcification, morphology, blood flow grade, and RI did not significantly differ between the responder or non-responder groups at pre-treatment and early-treatment phases. Although the tumor size may decrease when chemotherapy is effective, the regression pattern of tumor cells after NAC is non-concentric, but rather sporadic and multifocal. The size of the lesion may not significantly change, but the tumor cell density is significantly reduced, which results in errors in evaluating images (27, 28).

Radiomics is a relatively new technique that can excavate high-throughput digital features from digital medical images to quantify the heterogeneous characteristics of breast tumors, and analysis of this data may improve diagnosis, molecular typing, chemotherapy effect evaluation, prognosis analysis, and prediction (29, 30). Several previous studies have investigated the usefulness and reliability of MRI-radiomics models in predicting pathologic responses to NAC in breast cancer (31, 32). To the best of our knowledge, few reports have investigated the feasibility of using an US radiomics approach in breast cancer to predict the pCR of NAC, and most of these focus on pre-treatment (9). Pre-treatment baseline imaging is associated with primary tumor characteristics, while after-treatment images can directly reflect the response status, such as tumor cells becoming hypoxic and fragmenting after NAC, leaving fibrotic and collagenous tissues (33, 34). Therefore, we constructed a radiomics model by incorporating pre- and early treatment US information based on the combination of the original characteristics of the tumor and changes in the internal characteristics of the tumor after early treatment to accurately predict the response to chemotherapy, which differed from previous radiomic studies that involved only pre-treatment MRI or US data for pCR prediction. As shown in the results, RS1 (pre-treatment baseline) and RS2 (at early treatment of two cycles of chemotherapy) were independent predictors of NAC response and showed good performance, with AUCs of 0.811 and 0.722, respectively, and accuracies of 0.750 and 0.678 in the training groups, respectively. RS2 had significantly higher predictive performance than RS1, further demonstrating the important predictive value of early post-treatment US imaging. However, as US imaging is relatively dependent on the examining physician, it is difficult for the imaging parameters such as mass level, gray gain scale, and US energy before and after chemotherapy to be highly consistent. The effects of chemotherapy vary due to different molecular types and stages of tumors, resulting in different structural heterogeneity, and the residual tumor tissue and necrotic tissue after chemotherapy may affect the comparison of radiomic features. The above two factors may lead to the instability and randomness of differences in radiomic features before and after chemotherapy, which may result in the model with Delta RS with decreased prediction performance and lead to a decrease in the accuracy and repeatability of NAC prediction. In addition, the results of this study showed that the predictive performance AUC and accuracy of the delta radiomics were 0.714 and 0.692, respectively, in the validation sets, which did not achieve an ideal predictive value and had limited clinical predictive value. The above reasons are related to our decision to exclude Delta RS from multivariable modeling in this study.

Radiomic models are commonly established by a black-box approach, which is usually uninterpretable or difficult to interpret in the inner mechanisms of the model in the prediction process. We selected the parameters to be included in the radiomics model and the corresponding tumor characteristics. Radiomics features are usually difficult to intuitively interpret by the naked eye, but these can capture the heterogeneity and complexity of the tumor microenvironment. In this study, nine features in RS1 (pre-treatment baseline), 11 features in RS2 (early treatment), and eight features in Delta RS (baseline-early treatment) were finally selected as the most predictive factors by LASSO regression with 10-fold cross validation (**Table 2**). Most of the selected features are first-order features and texture features after wavelet transform. The median represents the median gray level intensity within the ROI. Kurtosis is a measure of the ‘peakedness’ of the distribution of values in the image ROI. A higher kurtosis implies that the mass of the distribution is concentrated towards the tail(s) rather than towards the mean. A lower kurtosis implies the reverse: that the mass of the distribution is concentrated towards a spike near the mean value. Skewness measures the asymmetry of the distribution of values about the mean value (35, 36). Texture parameters reflect specific positions relative to each other and capture subtle changes occurring within images to quantify intra-tumor heterogeneity by the gray-level co-occurrence matrix (GLCM) and gray-level run-length matrix (GLRLM) methods. Cluster prominence is a measure of the skewness and asymmetry of the GLCM. A higher value implies more asymmetry about the mean, while a lower value indicates a peak near the mean value and less variation about the mean. IMC2 assesses the correlation between the probability distributions of *i* and *j* (quantifying the complexity of the texture) (37, 38). Wavelet transform, which calculates the resolution of image signals in different time, space, and frequency scale planes, is useful for replaying even subtle but important texture information that is neglected by observers in low-contrast US images, so the texture features after wavelet transform are used to construct prediction models in many radiomic studies (39, 40). Besides, radiomics features ‘wavelet.LH_firstorder_Mean’, ‘wavelet.LHH_glcm_ ClusterProminence’,’wavelet.LHH_glcm_Imc2’, and ‘wavelet.HHH_firstorder_Kurtosis’ were significantly associated with response status in both pre- and early-treatment US images, implying that these features robustly reflect NAC response.

Next, we found that several clinical characteristics are correlated to NAC response, namely, molecular typing, PR, and Ki-67 expression. However, only Ki-67 expression similar to the RS1 and RS2 was a significant predictive indicator, with a moderate AUC of 0.643 after multivariate logistic regression analysis, and as expected, Ki-67 expression as a dependent predictor of NAC response in breast cancer has been proven in other studies (41, 42). Taking into account that clinical factors and radiomic features influence NAC responses, a nomogram that incorporated RS1, RS2, and Ki-67 state was developed, and the nomogram showed excellent ability to predict NAC response, with an AUC of 0.866 and accuracy of 0.785 in the validation cohort, achieving greater predictive efficacy than the other constructed model (**Figure 6**). Nomograms are simple tools for decision-making that have been widely used to predict medical prognosis and outcomes by combining multiple risk factors. Recently, a study (16) using radiomic nomograms to predict pCR of NAC in breast cancer based on the pre- and post-treatment US information of patients reported prediction performance that was slightly higher than ours. This may be related to the fact that the mass after complete chemotherapy is more reflective of the internal state of the tumor than the mass after early chemotherapy. This study focused on making a comprehensive prediction of the efficacy of the tumor after complete NAC and before surgery. Compared to that study, our greatest advantage is that NAC responses can be evaluated pre-treatment and early-trial-treatment, and there is no need to complete a full chemotherapy regimen for several months. Patients who are predicted to be non-responders could have a modified chemotherapy regimen, proceed directly to surgery, or investigate other treatment options. Early knowledge of patient response to chemotherapy allows early intervention and potential adaptation of a more personalized therapy.

This study has some limitations that should be acknowledged. First, due to patients’ own conditions, namely, breast density, mass size and location, and fat layer thickness, to obtain clearer imaging, each instrument parameters of the patient such as imaging depth, imaging focus, 2-D and color gain size were not consistent. Thus, it is hard to evaluate whether different parameters would affect the performance of the model. Second, the present study was a single-center research study. Although the nomogram has been evaluated to have a good predictive performance in an independent validation cohort, further additional investigations at other centers is necessary to assess the reliability of this prediction model.

In conclusion, breast US imaging at pre-treatment and early treatment (after completion of two cycles of chemotherapy) provides prognostic information on tumor response for NAC. We developed a NAC response prediction model based on pre-treatment and early treatment US imaging and significant clinical characteristics (Ki-67 expression) using a radiomics approach and obtained good predictive performance. Both nomogram and radiomics signature can be used as tools to assist clinicians in assessing NAC response in breast cancer patients, which can serve as an effective diagnostic reference for determination of NAC efficacy and timely guide treatment.

## Data Availability Statement

The raw data supporting the conclusions of this article will be made available by the authors, without undue reservation.

## Ethics Statement

The studies involving human participants were reviewed and approved by the Affiliated Cancer Hospital of Guizhou Medical University. The patients/participants provided their written informed consent to participate in this study.

## Author Contributions

MY and QDu contributed to the conception and design of the experiments. MY, HL, and QDa performed experiments, analyzed data, and wrote the manuscript. LY, SZ, ZW, and JL assisted in the writing and analyzed data. All authors listed have made a substantial, direct, and intellectual contribution to the work and approved it for publication.

## Funding

This work was supported by Science and technology Fund project of Guizhou Provincial Health Commission (No. gzwjkj2020-1-042) and Guizhou Science and technology support project, social development field ([2021]451).

## Conflict of Interest

Author HL was employed by GE Healthcare.

The remaining authors declare that the research was conducted in the absence of any commercial or financial relationships that could be construed as a potential conflict of interest.

## Publisher’s Note

All claims expressed in this article are solely those of the authors and do not necessarily represent those of their affiliated organizations, or those of the publisher, the editors and the reviewers. Any product that may be evaluated in this article, or claim that may be made by its manufacturer, is not guaranteed or endorsed by the publisher.
