# Supplementary material for: Treatment Response Prediction Using Ultrasound-Based Pre-, Post-Early, and Delta Radiomics in Neoadjuvant Chemotherapy in Breast Cancer
Source: Front Oncol. 2022 Feb 7;12:748008. doi: 10.3389/fonc.2022.748008 (PMC8859469; doi:10.3389/fonc.2022.748008)
Supplement: Supplementary file 1 [file DataSheet_1.docx]

## Supplement Material


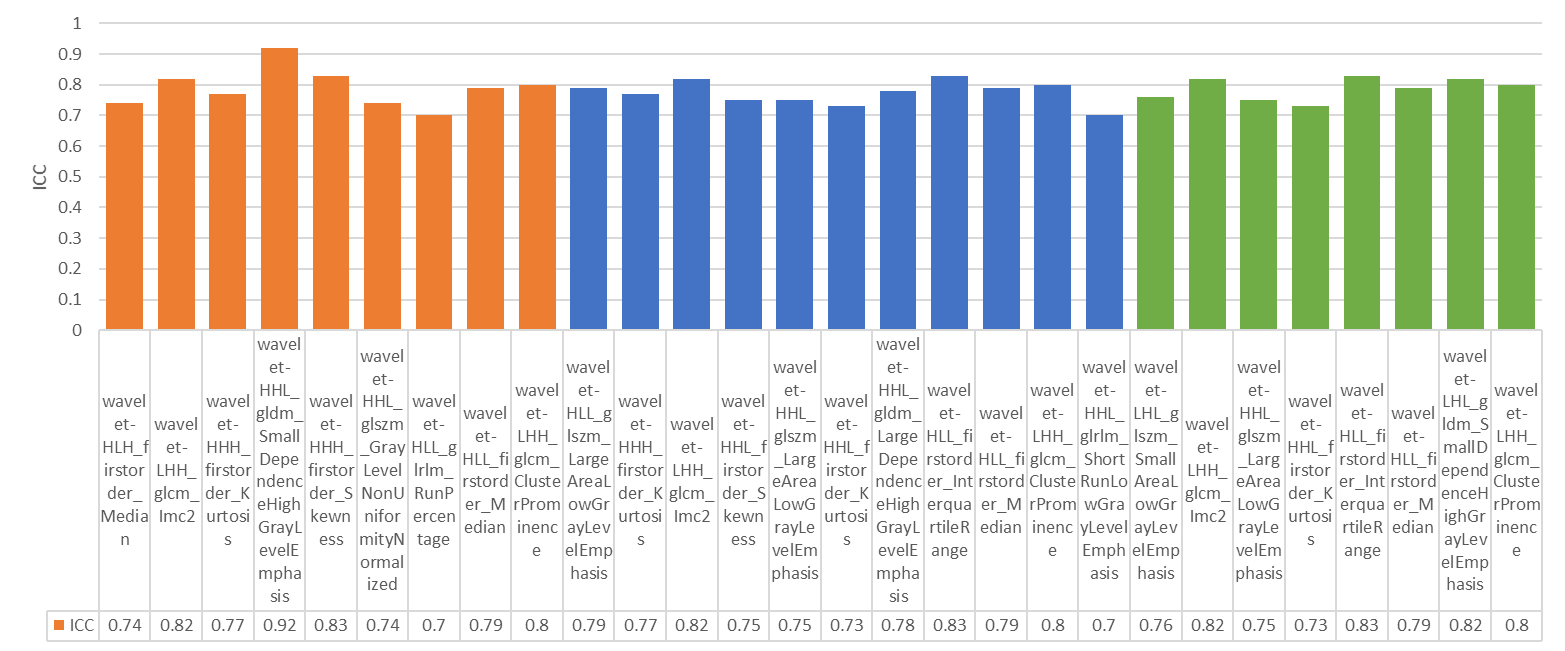


Figure S1 shows the specific values of ICC of each selected feature. represent in baseline model (RS1), represent in baseline model(RS2), represent in Delta RS.


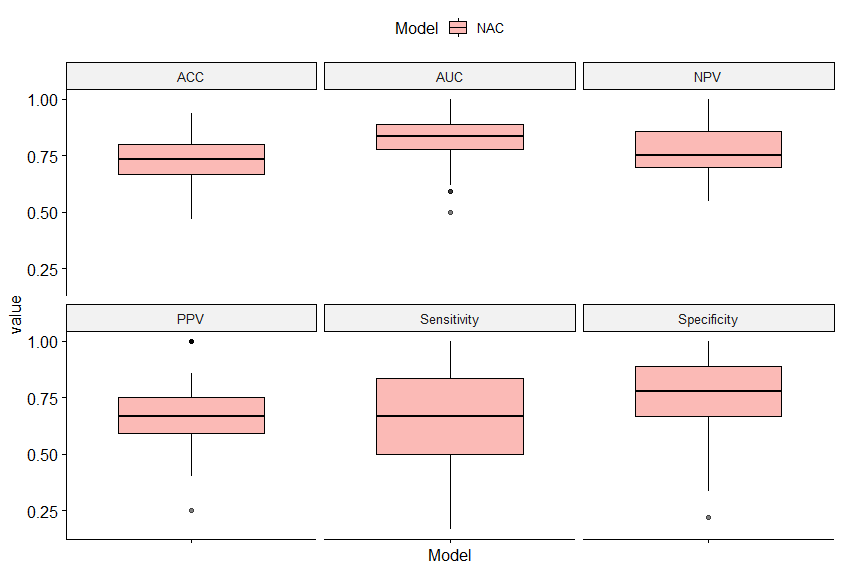


**Figure S2** shows the results of the ten-fold cross validation, which verifies the stability of the constructed model.
